# Biomarkers for Bronchopulmonary Dysplasia in the Preterm Infant

**DOI:** 10.3389/fped.2016.00033

**Published:** 2016-03-31

**Authors:** Lidys Rivera, Roopa Siddaiah, Christiana Oji-Mmuo, Gabriela R. Silveyra, Patricia Silveyra

**Affiliations:** ^1^Department of Pediatrics, The Pennsylvania State University College of Medicine, Hershey, PA, USA; ^2^Department of Biochemistry and Molecular Biology, The Pennsylvania State University College of Medicine, Hershey, PA, USA

**Keywords:** bronchopulmonary dysplasia, pulmonary hypertension, VLBW babies, biomarkers, preterm infants

## Abstract

Bronchopulmonary dysplasia (BPD) is a chronic inflammatory lung disease of very-low-birth-weight (VLBW) preterm infants, associated with arrested lung development and a need for supplemental oxygen. Over the past few decades, the incidence of BPD has significantly raised as a result of improved survival of VLBW infants requiring mechanical ventilation. While early disease detection is critical to prevent chronic lung remodeling and complications later in life, BPD is often difficult to diagnose and prevent due to the lack of good biomarkers for identification of infants at risk, and overlapping symptoms with other diseases, such as pulmonary hypertension (PH). Due to the current lack of effective treatment available for BPD and PH, research is currently focused on primary prevention strategies, and identification of biomarkers for early diagnosis, that could also represent potential therapeutic targets. In addition, novel histopathological, biochemical, and molecular factors have been identified in the lung tissue and in biological fluids of BPD and PH patients that could associate with the disease phenotype. In this review, we provide an overview of biomarkers for pediatric BPD and PH that have been identified in clinical studies using various biological fluids. We also present a brief summary of the information available on current strategies and guidelines to prevent and diagnose BPD and PH, as well as their pathophysiology, risk factors, and experimental therapies currently available.

## Introduction

Bronchopulmonary dysplasia (BPD) is a chronic inflammatory lung disease associated with arrested pulmonary development and a need for supplemental oxygen. According to the 2001 NIH Consensus Group, the clinical definition of BPD includes requirement of ventilatory support and supplemental oxygen at 36-week postmenstrual age (PMA) (1). Although the cause of the disease is multifactorial and includes both genetic and environmental factors, the mechanisms associated with the disease pathogenesis are not completely understood (2).

The incidence of BPD corresponds to 10,000 premature infants in the United States annually (3). The affected infants often have prolonged and recurrent hospitalizations that can lead to lifelong alterations in lung function, compromising their quality of life (3). The long-term consequences of BPD have been described in a variety of studies, and include altered pulmonary function, increased susceptibility to infection, and increased incidence of inflammatory lung disease and airway hyper-reactivity. In addition, approximately 40% of pediatric BPD patients develop pulmonary hypertension (PH), a serious debilitating disease associated with progressive elevation of pulmonary artery pressure that can lead to right ventricular failure and death (4).

In the recent years, PH has been associated with significant morbidity and mortality in infants with BPD (5, 6). A recent retrospective cohort study on PH patients indicated that the disease still presents with high morbidity, mortality, and cost of treatment (7). However, diagnostic biomarkers for predicting disease risk and standardized clinical criteria for PH diagnosis are lacking, there are currently not enough studies focusing on neonatal PH associated with BPD. Thus, it is important to continue to investigate the mechanisms underlying the pathogenesis of PH, and in particular, of BPD-associated PH, in search for alternatives that will allow for better therapeutic management and prevention strategies.

## Pathophysiology of BPD and PH

**Figure 1:**
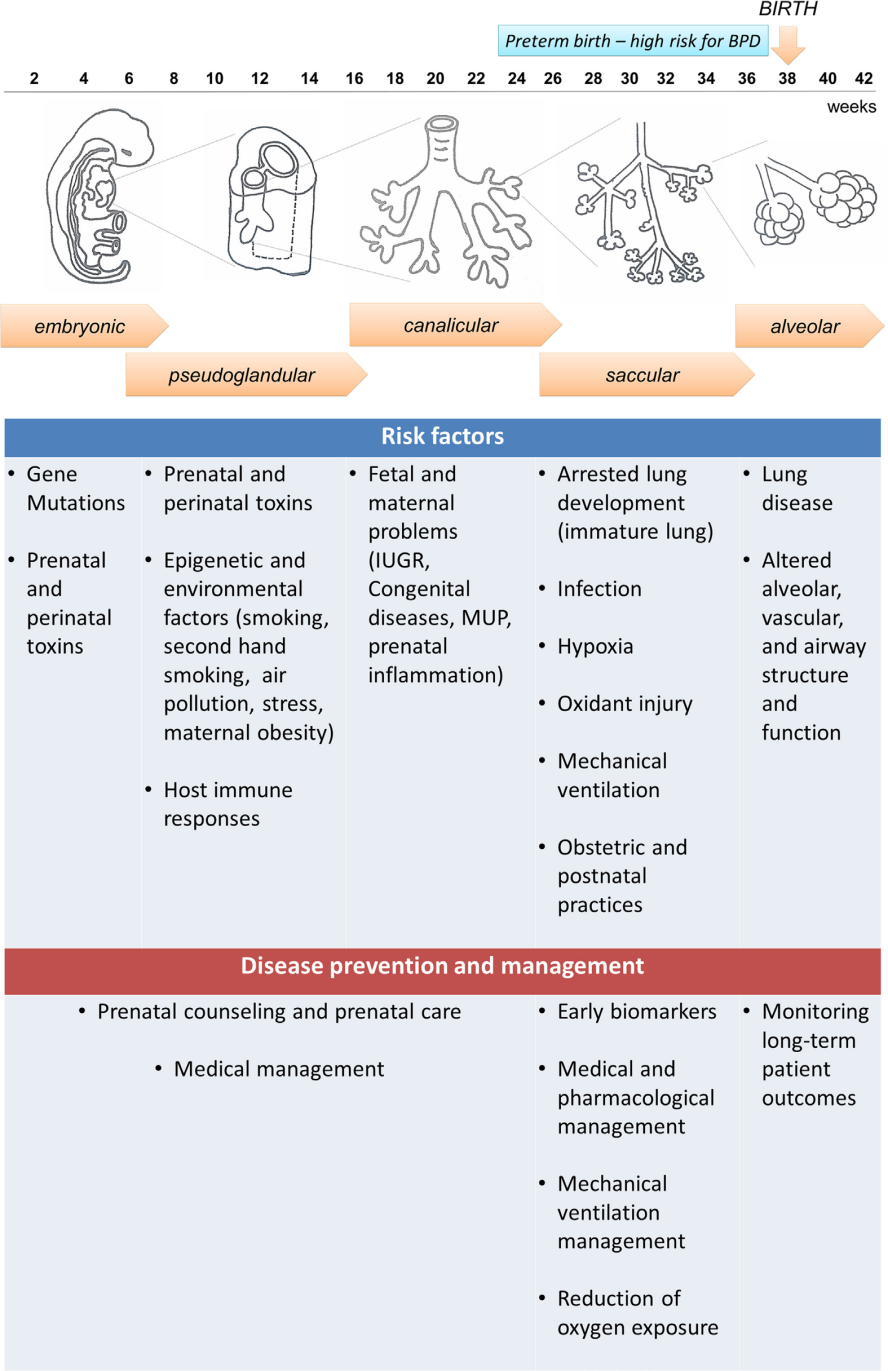
**Embryonic and fetal lung development and bronchopulmonary dysplasia (BPD): five stages are identified in human fetal lung development that can be affected by both antenatal and postnatal factors, resulting in increased susceptibility to develop BPD**. Preterm infants born at the canalicular or saccular stages have incomplete lung development and high risk for BPD. However, not all preterm infants develop BPD, indicating that other factors can contribute to the disease development, and the disease could be preventable. A summary of risk factors and potential prevention and management strategies is presented in the diagram.

To have a clear understanding of the pathophysiology of BPD, it is necessary to understand the normal lung development and the molecular pathways associated with the disease. Five stages have been identified in lung development: embryonic, pseudoglandular, canalicular, saccular, and alveolar (Figure 1). The first stage, named embryonic stage (4–7 weeks gestation), begins with the formation of a lung bud that emerges from the ventral foregut and posterior division to form two buds that lie on either side of the future esophagus. Successive branching within the expanding mesenchyme continues to form lobar and segmental airways in each lung. At the end of the embryonic stage, a network of nerves and ganglia are developed along with the smooth muscle to form the airways. These nerves and ganglia increased in size at 16 to 18 weeks gestation (8). Both the smooth muscle and mesenchymal cells secrete neurotrophic factors involved in both nerve growth and extension into the airways (9, 10). Next, during the pseudoglandular stage (7–17 weeks gestation), further division of the airway buds occurs, and there is a continuous differentiation of epithelial cells to form adult structures of cartilage, vascular networks, submucosal glands, and bronchial smooth muscle. At the following stage, the canalicular stage (17–27 weeks gestation), branching continues and respiratory airways form. In order to form the blood gas barrier, the epithelium starts thinning and becomes surrounded by a network of capillaries. Differentiation of the epithelial cells into type I and II pneumocytes occurs, and type II cells begin to produce surfactant. In this stage, sex differences begin to appear, with the female lung developing faster than the male lung, resulting in increased risk for BPD in prematurely born males than females (11, 12). In the saccular stage (24–36 weeks gestation) surfactant maturation continues, and the connective tissue forms thin septa within the airspaces containing a double pulmonary capillary layer (13, 14). Finally, in the alveolar stage, new tissue ridges lift off the existing primary septa and extend into the airspaces, where they subdivide the sacs into smaller alveoli (secondary septation) (15). This final stage begins at 36 weeks gestation and remains throughout infancy and adolescence.

The definition of BPD has evolved throughout the years (16). The “old BPD” definition was associated with the requirement of elevated oxygen levels and time spent on ventilatory support, which induced injury on an immature and surfactant deficient lung (17). This resulted in lung histopathological changes, damage of the airway epithelium, smooth muscle hyperplasia with areas of over inflation, atelectasis, fibrosis, and vascular hypertension (18). However, this definition has changed over time due to the utilization of antenatal steroids, aggressive medical interventions, and neonatal ventilation strategies that led to increased survival of extremely preterm infants. As a result, the “new” BPD is not primarily defined by increased ventilation and oxidant injury, but rather by the arrest of lung development (16). The lungs have some inflammation with accompanying pro-inflammatory cytokine response and fibrosis, but the saccular spaces show incomplete alveolar and vascular development (16, 19). Animal models including mechanically ventilated preterm baboons that developed BPD have shown arrest of alveolarization, decrease in the number of alveoli, and enlargement of airspaces that caused a reduction in the total lung internal surface (20).

Preterm and very-low-birth-weight (VLBW) infants have a higher risk of BPD because their lung development is often interrupted at the saccular and alveolar stages (3) (Figure 1). Therefore, these infants begin early pulmonary gas exchange with an inefficient alveolar and capillary network. In addition, multiple antenatal and postnatal factors, including infection, hyperoxia, and barotrauma can also contribute to the disease development, and can lead to the release of inflammatory cytokines that activate cell death pathways in the lung (21). Thus, BPD is the result of the healing and repair of lung damage that leads to fewer and larger alveoli, and a dysmorphic pulmonary vasculature (22). Furthermore, preterm infants are more vulnerable to oxidative stress due to their lack of adequate antioxidant protection, and their inability to induce antioxidant defenses while receiving supplementary oxygen (23). Pro-inflammatory cytokines are released in response to infection, but also in conditions of specific inflammation associated with mechanical ventilation (24). Inflammatory placental disorders and chorioamnionitis are also associated with BPD development due to coexistence of oxidative stress (25, 26).

It is still controversial whether BPD results in a decrease (27), or an increase (28) in pulmonary microvasculature. Studies in animal models and human tissue have shown alterations in both number and distribution of vessels in the distal lung (29). Studies in autopsies of infants who died from BPD have shown a decrease in the expression of the endothelial cell marker platelet/endothelial cell adhesion molecule-1 (PECAM-1), and vascular endothelial growth factor (VEGF), which are related to the dysmorphic and abnormal distribution of alveolar capillaries in thickened alveolar septa (14, 30). By contrast, high PECAM-1 levels were also observed in lungs of infants who died from BPD after being long-term ventilated, and associated with an increase in volume of air exchanging parenchyma and microvascular endothelial volume, but an immature microvasculature that resembled a saccular architectural pattern (31). Studies in mice also showed that the mRNA levels of VEGF (a known angiogenic factor) are significantly increased in type II pneumocytes during alveolar development. By contrast, VEGF absence in knockout mice resulted in delayed alveolarization and reduced capillary number in mouse lungs (32). Other reports have shown that low oxygen tension promotes growth and function of pulmonary artery endothelial cells during normal fetal lung development. In this regard, nitric oxide (NO) seems to play a critical role in the modulation of lung angiogenesis; thus, an impaired endothelial NO production may contribute to the abnormal lung vascular development seen in infants with BPD (33). On the other hand, angiotensin-converting enzyme (ACE) has been found in fetal tissue of human lungs as early as 12 weeks of gestation, with an increase of its levels throughout gestation and postnatally. However, ACE was downregulated in lung tissue from autopsies of infants who died from BPD, suggesting that ACE may be involved in the pathophysiology of BPD (34). Therefore, regardless of the definition, common findings (vascular remodeling, increased vascular tone, altered vasoreactivity, and decreased vascular surface area) are observed in patients diagnosed by the “new” and “old” BPD definitions, and both result in increased pulmonary vascular resistance and PH that can lead to right ventricular hypertrophy and dysfunction (4). Thus, abnormal vascular development in BPD leads to a decrease in the alveolar capillary surface area with inefficient gas exchange; which increases not only the need of prolonged ventilatory support, susceptibility for hypoxemia, and acute respiratory infections, but also to an increase in the risk of developing severe PH (35). Moreover, PH contributes to abnormal cardiopulmonary physiology with poor function of right ventricle (RV), impaired cardiac output, limited oxygen delivery, increased pulmonary edema, and risk of sudden death (35). Cardiac catheterization studies have revealed an abnormal pulmonary vascular physiology with abnormal vasoreactivity and elevated pulmonary vascular resistance such that even mild hypoxia results in marked elevation of pulmonary vascular pressures (35–37).

**Figure 2:**
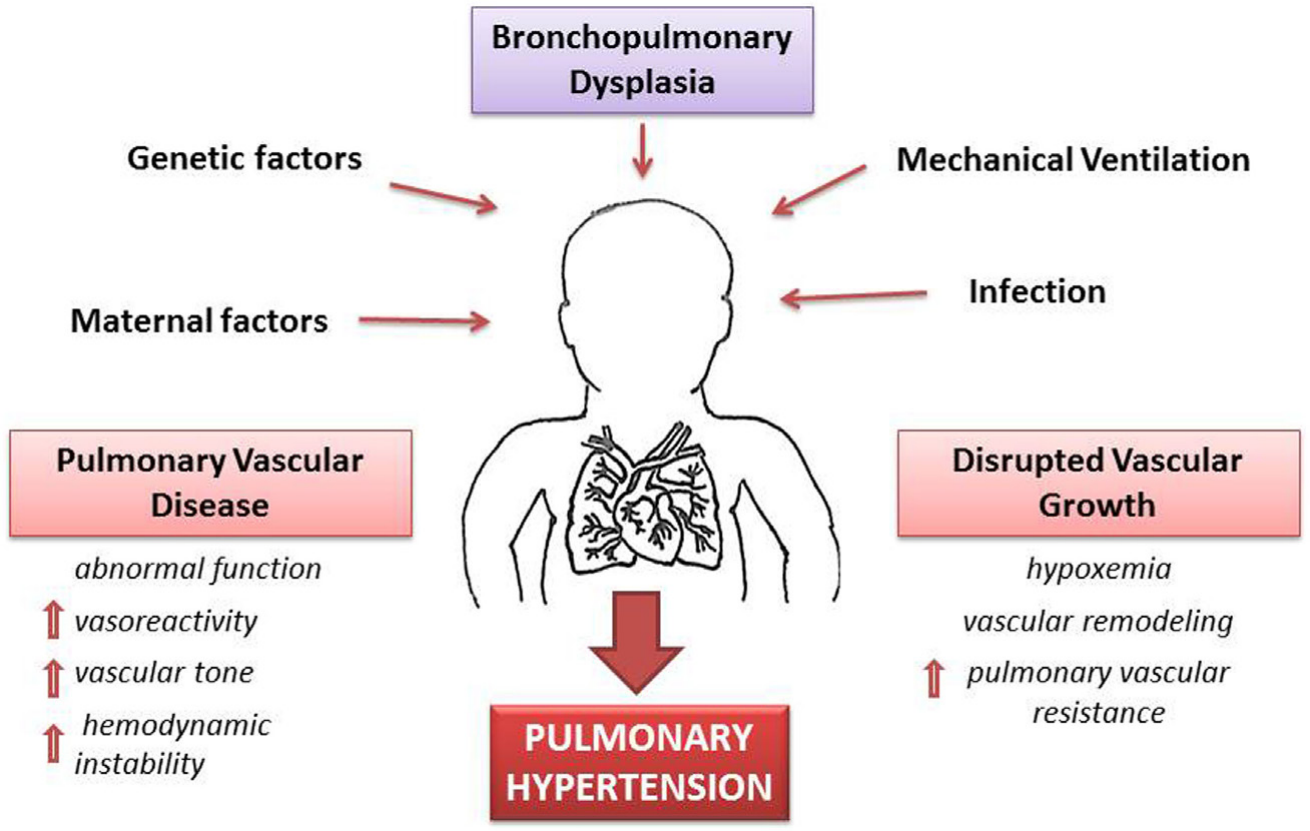
**Pathophysiology of pulmonary hypertension (PH) secondary to bronchopulmonary dysplasia (BPD)**. A combination of antenatal and postnatal factors leads to the development of bronchopulmonary dysplasia (BPD). Disruption of vascular growth is caused by hypoxemia and vascular remodeling with posterior increase of vascular resistance. These changes lead to pulmonary vascular disease with increases vasoreactivity, vascular tone, and pulmonary vascular resistance. Thus, development of PH is a multifactorial consequence of BPD, which results in hemodynamic instability and long-lasting health consequences.

A complex signaling between the developing lung epithelium and the adjacent vascular endothelium results in normal development of lung vasculature. These signaling pathways involve molecules, such as VEGF and endothelial nitric oxide (eNO), which in animal models have been found to increase progressively during pulmonary vascular growth in the last half of gestation (38). Other preliminary studies have demonstrated a pharmacological inhibition of VEGF receptors that leads to attenuation of vascular growth and abnormal vasodilator capacity of pulmonary blood vessels (39, 40). This increased pulmonary vascular vasoreactivity leads to increased overall vascular resistance that is exaggerated even with minor left to right shunt of blood via patent ductus arteriosus (PDA), cardiac (patent foramen ovale, arterial receptor defect, ventricular septal defect) or intrapulmonary arteriovenous pulmonary vessels that results in a marked vasoconstrictor response and greater hemodynamic instability (Figure 2).

## Clinical Classification of BPD and PH

According to the National Institute of Child Health and Human Development (NICHD) and National Heart, Lung, and Blood Institute (NHLBI) Workshop criteria, BPD is defined in three levels of severity, according to gestational age (GA) and oxygen requirement of preterm infants. *Mild* BPD is defined as requiring at least 28 days of supplemental oxygen therapy plus discharge or termination of supplemental oxygen therapy by 36 weeks PMA (infants <32 weeks GA), or by 56 days postnatal age or discharge (infants ≥32 weeks GA). *Moderate* BPD is defined as requiring at least 28 days of supplemental oxygen therapy with less than 30% of oxygen at 36 week PMA (infants <32 weeks GA) and at 56 days postnatal age or discharge (infants ≥32 weeks GA). Finally, *severe* BPD is defined as requiring at least 28 days of supplemental oxygen therapy with 30% of oxygen, greater or positive pressure at 36 weeks PMA or at discharge (for infants <32 weeks GA), and at 56 days postnatal age or discharge (for infants ≥32 weeks GA) (1).

The clinical diagnosis of pediatric PH can be done by two different definitions: the WHO and the Evian classifications. We have summarized the major differences between the two classification systems in Table 1. In the WHO categorization, the criteria used for pediatrics PH are similar to that of adult PH and includes multiple factors (41). By contrast, the Evian Classification categorizes PH into five classes: (1) pulmonary arterial hypertension, (2) PH due to left heart disease, (3) PH associated with pulmonary diseases/chronic hypoxemia, (4) chronic thromboembolic PH, and (5) PH with unclear multifactorial mechanisms (42, 43). This classification has contributed to the understanding of the disease pathophysiology, treatment, and long-term consequences for adult patients. Nevertheless, exclusive disorders presented in early childhood are not included or defined, which represents a concern for the management of clinical strategies in infants. As a result, the pulmonary vascular research institute (PVRI) in Panama considered specific points for the pediatric population, and classified the disease into 10 categories that help understand aspects of the impaired structural and functional adaptation that occurs during transition from fetal to neonatal circulation, as well as their association with prenatal, perinatal, and chromosomal influences (44). However, although the PVRI Panama classification has a multifactorial and clinical approach that focuses on the understanding of the disease and improvement of its diagnosis, more studies are needed before it could be used in the clinic, and the WHO classification continues to be preferred (35).

**Table 1:** **Comparison of pediatric pulmonary hypertension classification systems**.

WHO clinical classification system	Panama classification system
Understanding of congenital heart disease in adult survivors	Based on clinical practice
	Not intended to be used as a therapeutic guide
Does not include perinatal maladaptation, maldevelopment, and pulmonary hypoplasia as causative factors in neonatal pulmonary hypertension	Emphasizes concepts of prenatal and perinatal maladaptation, maldevelopment, and pulmonary hypoplasia
	Unique to pediatric disorders (neonates to adolescents)
	Gives importance to chromosomal and genetic syndromes

## Risk Factors

Although BPD and PH have similar risk factors, including extremely low GA at birth, oligohydramnios, duration of mechanical ventilation, excessive oxygen therapy, and infection (45–48), the two diseases do not always present together. Moreover, there is no clear association of BPD severity with PH susceptibility, since clinical reports show that some infants with severe BPD do not develop PH, whereas others with mild BPD develop PH (48, 49). Histopathological analyses have demonstrated that placental maternal vascular underperfusion (MVU) in high-risk pregnancies has also been associated with increased risk of BPD and PH (50). Recently, a study conducted in a sheep model of chronic fetal hypoxia showed that fetal stressors (*in utero* hypoxia and ischemia) can affect pulmonary vascular growth, and can lead to uteroplacental insufficiency, and perhaps contribute the BPD and PH development (51). Preeclampsia has been recognized as a risk factor for BPD, and rat experimental models of BPD have identified possible mechanisms associated with this increased risk. For example, overproduction of soluble VEGF receptor-1 (VEGFR-1, also known as sFlt-1), which antagonizes VEGF reducing its free circulating levels, seems to play a role in the development of BPD in newborns of mothers who suffer from preeclampsia. An increase in sFlt-1 in amniotic fluid has been associated with an impairment of alveolarization and pulmonary vascular growth, as well as biventricular hypertrophy in newborns, which suggests pulmonary and systemic hypertension (52). Furthermore, sex differences in lung development have been reported, and indicated that the developmental transcriptome differs in the lungs of males and females (11). In early development [i.e., before 96 days post conception (dpc)], the male lung is more mature than the female lung, but in late development (after 96 dpc), the male lung is less mature compared to the female lung (Figure 1). These differences do not seem to affect early fetal lung development, or impact BPD susceptibility at earlier times (53). However, later in lung development, females appear to be protected for a number of conditions, due to the production of estrogens, which modulate VEGF and platelet-derived growth factor (PDGF) expression (54). Therefore, males are more affected by extreme prematurity and associated morbidities (55). Experimental studies in mice suggest that the protection of estrogens remains until puberty, and studies in mice treated with estrogens showed a higher expression of anti-inflammatory factors and less risk of asthma development (56). Nevertheless, circulating estrogen levels appear to be associated with increased risk of PH in women adults. Recently, a study found high levels of estradiol and higher estradiol/testosterone ratio, as well as low DHEA (dehydroepiandrosterone) levels to be associated with an increased risk to develop PH development in men, indicating an abnormal increase of aromatization in men with PH (57).

On the other hand, studies focusing on the pathology of BPD have centralized research efforts toward genetic studies, identification of pathways of immunity, inflammation, and antioxidant defenses, mechanisms of vascular and lung remodeling, and surfactant protein biology (58). In this regard, genetic variants of surfactant proteins have been largely associated with risk for neonatal disease (59). A variant of the SP-A1gene (6A^6^) was identified as a risk factor for BPD in a German population, and two single-nucleotide polymorphisms (SNPs) in the SP-A2 and SP-D genes (1A^2^-rs2243639; 1A^2^-rs721917-rs2243639) were found as protective factors for BPD in a Greek population (59). Finally, studies aimed to predict the risk of BPD or death in preterm infants evaluated the score for neonatal acute physiology (SNAP). The SNAP is a physiological severity index for neonatal intensive care, based on 28 clinical items measured during the first 24 h of life. A high SNAP score was found associated with development of BPD or death in critical ill preterm neonates (60).

## Screening and Diagnosis of BPD and BPD-Associated PH

Due to the high incidence of PH in BPD patients, and its high mortality rate, it is important to identify novel methods to improve early detection of PH, so that timely preventive interventions and treatment that is more aggressive can be initiated to improve survival. The available strategies for disease management and prevention include controlled respiratory support, cardiac medications, vasodilators, and surgical or interventional cardiac catheterization procedures (35). The diagnosis of PH can be very difficult because most of its symptoms overlap with those of BPD (4). According to the most recent guidelines from the American Heart Association and the American Thoracic Society, echocardiograms must be performed in preterm infants with severe respiratory distress syndrome (RDS) undergoing mechanical ventilation, as well as in infants without improvement of RDS symptoms (35). Furthermore, patients who are diagnosed with moderate or severe BPD at 36 weeks of GA should be indicated echocardiogram screening for PH. Electrocardiogram is not recommended as screening of PH because it has very low sensitivity. Additionally, catheterization is also recommended in patients diagnosed with PH and BPD, especially if severe cardiorespiratory symptoms persist despite optimal management, or long-term PH pharmacotherapy (35).

## Long-Term Consequences of BPD and PH on Lung Health

Various clinical studies have explored the long-term consequences of BPD, particularly in adult patients with respiratory comorbidities. Some of these long-term consequences were evaluated in a study of patients born with BPD, RDS, preterm patients with no neonatal lung disease, and healthy patients born at term. The results of this study showed that patients born with BPD had worse pulmonary function and physical activity scores than patients in the other groups. These patients presented airflow obstruction, more gas trapping, decreased diffusion capacity, and evidence of bronchial hyper-reactivity when challenged with the methacholine test (61). Another study evaluated the metabolomic profile in exhaled breath condensates (EBC) of 20 adolescents with history of BPD and 15 healthy adolescents (controls). The results showed alteration of the lipid profile in EBCs from BPD survivors. One of the metabolites identified was lysophosphatidylcholine, an oxidized phospholipid known to induce superoxide production in neutrophils and platelet-activating factor (PAF) during inflammation. In addition, the investigators found abnormal levels of unsaturated phosphatidylserine, plasmenyl-phosphatidylserine, and surfactant phospholipids in the BPD survivors group, indicating that the lungs of these patients keep long-term metabolic abnormalities that conduce to inflammation and hyper-reactivity (62). On the other hand, studies in mouse pups exposed to hyperoxia demonstrated structural changes during lung development that affected their postnatal lung function, with more effects in males than in females. Furthermore, these mice showed increased susceptibility to viral infections, especially to influenza A virus that led to higher mortality and production of lung collagen (63).

## Biomarkers

As mentioned above, BPD is a multifactorial disease that remains a significant cause of mortality and morbidity in the preterm infant. Several studies conducted in patients and animals have identified a variety of biomarkers for BPD susceptibility that bear the potential to make early diagnosis and allow prevention of the disease progression, avoiding or decreasing its effects and sequelae in children and adults. To this date, biomarkers have been detected in several biofluids, including blood, urine, serum, tracheal aspirates, and lung lavage. This information, combined with data obtained from genetic and epigenetic studies, will contribute to the development of more effective diagnostic tools, to the discovery of molecular pathways associated with the disease development and progression, and to the identification of novel therapeutic targets. In the sections below, we have summarized the main findings of clinical studies conducted to seek biomarkers for BPD in various biological fluids.

### Biomarkers for BPD

#### Biomarkers in Blood

As indicated in Table 2, the vast majority of biomarkers identified in blood are of protein nature, and these represent molecules associated with immunity, inflammation, and oxidative stress. We have reviewed the literature to find studies identifying these biomarkers in various populations, and we have compared the available information on patient sample size, disease phenotype, classification criteria, sensitivity, specificity, and predictive value of each biomarker. In the sections below, we have grouped this information by biomarker or groups of biomarkers, in order to facilitate comparisons among studies.

**Table 2:** **Biomarkers for BPD and PH**.

Disease	Biomarker(s)	Specificity (%)	Sensitivity (%)	*p*-Value	AUC	Reference
BPD	CC16	82.6	85.7	<0.01	0.913	(64)
BPD	KL-6 (1 week)	87	79	<0.05	0.8307	(65)
BPD	KL-6 (2 weeks)	82	84	<0.05	0.8995	(65)
BPD	NGAL	85	94	<0.01	0.91	(66)
BPD	ETCO	92	80	0.05	0.96	(67)
BPD	ETCO	75	97	<0.01	0.97	(68)
CLD	ETCO	72	100	<0.05	0.87	(69)
BPD	CO-Hb	75	88.9	0.002	0.882	(70)
BPD	suPAR	84.6	88	<0.001	0.902	(71)
BPD	AFP	–	–	–	–	(72)
BPD	hCG	–	–	–	–	(72)
BPD	uE3	–	–	–	–	(72)
BPD	MMP-9	–	–	0.057	–	(73)
BPD	IL-6	86	51	0.003	0.849	(73)
BPD	gp130	82	51	0.048	–	(73)
BPD	PLGF	95	53	<0.001	–	(74)
BPD	Endostatin	–	–	0.029	–	(75)
BPD	NT-pro-BNP	–	–	0.006	–	(76)
BPD	BNP	85.7	76.1	0.028	0.8	(77)
BPD	rs3771150 (IL-18RAP)	–	–	0.012	–	(78)
BPD	rs3771171 (IL-18R1)	–	–	0.07	–	(78)
BPD	rs1245560 (SPOCK2)	–	–	–	–	(79)
BPD	rs1049269 (SPOCK2)	–	–	–	–	(79)
BPD	miR-133b, miR-7, miR-152, and miR-30a-3p	80	87	<0.01	0.91	(80)
BPD	miR-206	–	–	<0.01	–	(81)
BPD	miR-219	–	–	–	–	(82)
BPD	F2-isoprostane	–	–	0.53		(83)
BPD	8-OHdG (day 3)	62.9	82.4	0.037	0.037	(84)
BPD	8-OHdG (day 7)	61	85.7	0.02	0.770	(84)
BPD	β-2-microglobulin	83	73	0.0001	0.800	(85)
BPD	myoinositol	–	–	–	–	(86)
BPD	lactate	–	–	–	–	(86)
BPD	taurine	–	–	–	–	(86)
BPD	TMAO	–	–	–	–	(86)
“New” BPD	IL-6	–	–	0.11	–	(87)
“New” BPD	IL-8	–	–	0.25	–	(87)
“New” BPD	IL-10	–	–	0.15	–	(87)
“New” BPD	PDGF	–	–	0.42	–	(87)
“New” BPD	VEGF	–	–	0.67	–	(87)
“New” BPD	TGF			0.38	–	(87)
“New” BPD	MCP	–	–	0.93	–	(87)
BPD-PH	ADMA	–	–	0.02	–	(88)
BPD-PH	rs2781666 (arginase I)	–	–	0.047	–	(89)

##### KL-6, CC16, and NGAL

A systematic review of more than 500 articles identified nine studies that proposed serum Krebs von den Lungen (KL-6), Clara cell secretory protein (CC16), and neutrophil gelatinase-associated lipocalin (NGAL) as extremely good predictors for early BPD diagnosis (90).

Krebs von den Lungen, also known as MUC1, is a glycoprotein preferentially expressed and secreted by type II pneumocytes and bronchial epithelial cells, whose expression has been correlated with the presence and severity of several lung diseases (91, 92). In a prospective study, cord blood (CB) and plasma KL-6 were identified as potential biomarkers for BPD. The study included 135 preterm infants with a GA <32 weeks; divided into two groups: GA < 28 weeks (*n* = 42) and GA > 28 weeks (*n* = 93). Plasma KL-6 levels of at least 199 U/mL at 1 week of life, or 232 U/mL at 2 weeks of life were excellent predictors of moderate/severe BPD at <28 weeks GA (Table 2) (65).

Clara cell secretory protein, also known as CC10 or CCSP, is a protein secreted by the tracheobronchial epithelium that has been identified as a marker for lung epithelial injury (93). A study comparing blood CC16 levels in mechanically ventilated neonates identified higher CC16 serum concentrations in infants who developed BPD as early as 2 h after birth (64). Elevated serum levels of CC16 at 72 h after birth were highly predictive of BPD development (Table 2).

Neutrophil gelatinase-associated lipocalin, also known as lipocalin-2, is a glycoprotein expressed by granulocytes whose expression has been found associated with respiratory and inflammatory disease (94). In a study of 36 preterm infants, serum NGAL levels of ≥82 ng/mL at birth were predictors of BPD in patients with GA < 31 weeks (66).

##### ETCO and CO

A number of studies have assessed end tidal carbon monoxide (ETCO) as a good biomarker for BPD (67, 90). Sequential ETCO measurements in a population of preterm infants (*n* = 50) born at GA < 32 weeks revealed higher ETCO levels in infants who develop BPD (*n* = 14). ETCO levels of >2.15 ppm at day 14 after birth were potential predictors of BPD (Table 2) (67). A second study by the same group evaluated 78 preterm infants with GA < 33 weeks, classified into mild BPD (*n* = 12), moderate BPD (*n* = 15), severe BPD (*n* = 12), and no BPD (*n* = 39). ETCO at day 14 was a good predictor of BPD with high sensitivity and specificity (68) (Table 2). Another study evaluated associations of ETCO levels with severity of RDS and chronic lung disease (CLD), and found that infants with RDS had significantly higher ETCO levels during the first week of life than controls (69). Moreover, infants who developed CLD had higher ETCO levels (>2.5 ppm) during the first 12 h of life than those without CLD (Table 2).

Since carbon monoxide (CO) is produced endogenously during oxidative stress processes and pro-inflammatory cytokine release (95, 96); a study assessed the potential of plasma CO as a biomarker for BPD using carboxyhemoglobin (CO-Hb) as a measure of CO in the blood. This study identified significantly higher CO-Hb plasma levels in infants with moderate/severe BPD vs. no/mild BPD (*n* = 25) (Table 2), and concluded that CO-Hb could be used as a biomarker of oxidative damage and inflammation in the lung of preterm infants (70).

##### sST2, IL-33, and suPAR

A group of investigators studied the potential of three soluble biomarkers for BPD prediction in umbilical CB and in peripheral blood (PB): soluble ST2 (sST2), interleukin 33 (IL-33), and soluble urokinase plasminogen activator receptor (suPAR). The ST2 gene produces a soluble secreted form (sST2) and a transmembrane form (ST2L) of the IL-1 receptor in Th2 cells, mast cells, and macrophages (97). Interleukin 33 (IL-33) is a member of IL-1 cytokine family and a modulator of inflammation in the pathogenesis of chronic diseases (98). The urokinase plasminogen activator (uPA) and its receptor (uPAR) are expressed in endothelial and immune cells and participate in the regulation of cell migration, angiogenesis, proliferation, and adhesion. A soluble form of uPAR (suPAR) can be found and measured in body fluids, such as plasma and urine (99). The study evaluated concentrations of IL-33, sST2, and suPAR in a cohort of 38 preterm infants (GA ≤ 32 weeks) with and without BPD, and found significantly higher levels of PB-suPAR and PB-sST2 in BPD patients (Table 2), but no significant CB levels of these markers. Patients with PB-suPAR levels >1.55 ng/mL had higher risk for BPD development (71).

##### IL-6, IL-6R, gp130, and MMP-9

An independent study conducted in CB from 134 premature infants evaluated the potential of interleukin-6 (IL-6), its receptor IL-6R, soluble gp130, and matrix metalloproteinase 9 (MMP-9) as biomarkers of BPD. IL-6 is a cytokine involved in inflammation, immune regulation, hematopoiesis, and oncogenesis (100). IL-6 binds to IL-6R, which associates with the signal transducing membrane protein gp130 (a molecule that has a membrane and a soluble form), with a consecutive activation of downstream signaling cascades that result in gene expression regulation (101–103). MMP-9 acts as a defense factor against lung damage and inflammation in animal models (104). The study found increased levels of these biomarkers in the BPD group, with higher levels of IL-6 and soluble gp130 correlating with highest risk for BPD (Table 2). Levels of IL-6 of 46.125 pg/mL were good predictors of BPD in infants with GA < 28.5 weeks (73). However, a separate study comparing serum of patients diagnosed with the “old” vs. “new” BPD definition found increased levels of profibrotic/angiogenic factors [transforming growth factor-beta1 (TGFβ-1); PDGF BB fraction, PDGF-BB, and VEGF] and decreased levels of IL-6 in “new BPD” patients, but no differences in the “old BPD” patients (87).

##### VEGF, PLGF, and BNP

We mentioned earlier that VEGF plays an important role in alveolar development, and that altered levels of VEGF could constitute risk factors for BPD and PH development. A research group examined levels of placental growth factor (PLGF, a member of VEGF family involved in angiogenesis) in CB of BPD patients vs. controls. The study found higher levels of PLGF (>17 mg/dl) in CB of BPD patients (Table 2); however, the concentration of PLGF was also associated with the number of days on ventilator (74). Another biomarker related to VEGF is endostatin, which antagonizes the effects of VEGF and other pro-angiogenic factors (105). A study evaluating the concentration of endostatin in cord plasma of 92 preterm infants (GA < 32 weeks; BW < 1500 g) and 48 healthy term infants (GA > 37 weeks; BW > 2500 g) found that endostatin concentration in VLBW infants was significantly lower than that in healthy term infants. However, during the first two postnatal weeks, the levels of endostatin were high in VLBW preterm infants if these were intubated and receiving high concentrations of oxygen. Moreover, in an independent study, higher endostatin levels were found in VLBW preterm infants who developed BPD vs. those who did not (75).

Finally, due to the association of BPD with diastolic dysfunction and impaired left ventricle myocardial performance index in preterm infants (106), some studies have identified a significant correlation of B-type natriuretic peptide (BNP) with BPD diagnosis. In one study conducted in infants who developed BPD at 4 weeks of age, plasma concentrations of *N*-terminal pro-BNP (NT-pro-BNP) were significantly increased in the BPD group (3208 pg/dl) vs. the control group (1122 pg/dl). However, one problem with this study was that the levels of NT-pro-BNP were much higher in the control group than the levels previously reported in full term healthy neonates (76). In a different study, the potential of BNP as a biomarker for BPD severity was assessed in 60 premature infants (GA < 32 weeks). The study revealed higher plasma BNP levels in moderate and severe BPD patients at GA = 36 ± 2 weeks or at discharge time. As a result, BNP plasma levels of 24.4 pg/mL at GA = 36 weeks were proposed as a predictive biomarker for BPD severity (77).

##### Serum Biomarkers in Maternal Blood

In a large study (662,889 pregnancies), a research group examined the relationship between second-trimester maternal serum levels of alpha-fetoprotein (AFP), human chorionic gonadotropin (hCG) and unconjugated estriol (uE3) and BPD. The results showed that increased levels of AFP, hCG, and uE3 were associated with an increased risk of BPD in the newborn. Risk for BPD was higher when AFP and hCG levels were above the 95th percentile and when uE3 levels were below the fifth percentile (72).

In summary, the vast majority of biomarkers for BPD have been studied in serum. The advantages of using serum are the availability of reference protein values for normalization, and the relatively high concentrations of some of the biomarkers identified. The disadvantages of using serum are related to challenges associate with sample collection and volumes needed, especially in VLBW newborns, which could explain why most studies were conducted in very small sample sizes. Of the protein biomarkers studied, the following showed very high sensitivity to identify infants at risk for BPD: NGAL, KL-6, IL-6, suPAR, and BNP. However, the most sensitive markers were PLGF and ETCO (Table 2). We believe that non-protein molecules [e.g., EtCO and microRNAs (miRNAs)] are among the most promising biomarkers to be used in future studies, because of the nature of the techniques used for their detection and quantification (EtCO is measured by the use of an electrochemical sensor, and miRNAs can be amplified by PCR and/or RNA sequencing). Moreover, the diagnostic and prognostic potential of miRNAs as biomarkers relies mainly on their high stability even after being subjected to severe conditions (e.g., extreme temperatures, very low or high pH, extended storage, and numerous freeze–thaw cycles) that would normally degrade most RNAs (107). Furthermore, detecting specific miRNA species is generally much easier and accessible than detecting specific proteins (28). The development of more sensitive and specific antibodies for protein biomarkers may also favor the use of these in the clinic. A combination of biomarkers that are easy to obtain and measure in small amounts of serum will provide a useful tool for BPD diagnosis, especially when dealing with low sample volumes in VLBW infants.

#### Biomarkers in Urine

Over the past few years, the study of metabolomics (low molecular mass metabolites) in biological samples has been relevant in the process of phenotypic description of neonatal diseases (108, 109). A study evaluating metabolite levels in urine samples collected at 24–36 h post birth in 36 preterm infants found increased levels of four metabolites: lactate, taurine, trimethylamine N oxide (TMAO), myoinositol, and decreased levels of gluconate in BPD vs. non-BPD patients. Based on these results, it was suggested that an increase in anaerobic respiration occurs during the course of BPD, as indicated by the increase in lactate. TMAO and taurine participate in processes of osmoregulation and membrane stabilization, and taurine has known roles in detoxification, apoptosis, and calcium homeostasis functions. Interestingly, the investigators concluded that BPD is a congenital disease associated with genetic factors and intrauterine epigenetics issues (86).

One very successful biomarker evaluated in urine of BPD patients is β-2-microglobulin (B2M), due to its association with human histocompatibility antigen (HLA) in inflammatory diseases (110, 111). A study conducted in 96 preterm infants found higher B2M urine levels in BPD patients in the first 48 h of birth, indicating that B2M could serve as a biomarker for BPD (85).

With oxygen toxicity as significant contributor of BPD pathophysiology, some studies aimed to identify metabolite products of oxidant injury in the urine. In one study, F2-isoprostane (F2-IsoP), a metabolite formed by lipid peroxidation of cellular membranes, was measured in 40 preterm infants with and without BPD, but found no significant of F2-IsoP in the neonatal period (83). In another study, leukotriene E4 (LTE4) and 8-hydroxyguanosine (8-OHdG), two oxidant injury markers, were evaluated in the urine of 60 preterm infants. LTE4 results from the degradation of cell membranes, and it has been studied as a severity marker of asthma (112), and 8-OHdG is a biomarker of DNA damage (113). The results showed increased levels of 8-OHdG, but not LTE4, in moderate/severe BPD at day 7, and higher LTE4 levels in patients diagnosed with atypical BPD. According to these results, it was suggested that oxidative DNA damage is associated with the pathogenesis of classic BPD, and that inflammatory processes may have a more predominant role in atypical BPD (84).

Finally, the system of pulmonary neuroendocrine cells (PNEC), such as solitary cells and innervated clusters, and neuroepithelial bodies (NEB) produce a variety of neuropeptides (bombesin, calcitonin) and amines (5-HT). The role of these specialized cells is now beginning to be appreciated in normal lung but also in pulmonary diseases, such as BPD (114). Therefore, it has been proposed that the neuropeptides secreted by this system could potentially serve as biomarkers for neural damage associated with BPD. A research study conducted in premature baboons identified high urine levels of bombesin-like peptide (BLP) associated with BPD development, and suggested a role of BLP in mechanisms of lung injury. Furthermore, when the research group administered an anti-BLP blocking antibody to the BPD baboons, an improvement in the oxygenation index and a decrease of inflammatory cells were found, indicating that BLP could be an early biomarker for BPD and a potential therapeutic target (115).

In summary, urine biomarkers have the advantage of non-invasive procurement for large amounts, and relatively high protein stability when compared to other fluids. However, urine also has the disadvantage of wide variability in protein concentration, and lack of information on premature newborn’s urine protein profiles that will allow for easy normalization of biomarker expression. The studies described above indicate that β-2-microglobulin is a promising biomarker with high sensitivity and specificity that can help in early detection of BPD in newborns.

#### Biomarkers in Bronchoalveolar Lavage Fluid

In order to better understand cellular and molecular events that occur locally in the lung during the course of BPD, a research group evaluated protein changes in bronchoalveolar lavage fluid (BALF) from prematurely born infants with BPD. The study found that patients with lower GA (23–25 weeks) had increased levels of annexin-3, surfactant-associated protein A2 (SFTP-A2), and serum proteins, such as albumin, serotransferrin, and clusterin. These patients also had decreased levels of calcium signaling-related proteins, leukocyte elastase inhibitor, chloride intracellular channel (CLICL1), and calcyphosine (CAPS) when compared to patients with higher GA (26–29 weeks). By contrast, BALF measurements in BPD vs. non-BPD patients revealed higher CAPS, and lower calcium and integrin binding protein-1 (CIB1) levels in severe vs. mild BPD patients (116). CAPS is associated with the regulation of cell proliferation, cell differentiation, and surfactant secretion. Calcium signaling-related proteins regulate epithelial differentiation and development of the air–blood barrier. SFTP-A2 has known regulatory roles in surfactant phospholipid secretion; and both SFTP-A2 and annexin-3 are inhibitors of phospholipase A2 (PLA2), which is implicated in inflammation and surfactant catabolism. Therefore, in BPD, CIB1 downregulation might impair calcium signaling in lung cells, contributing to lung dysfunction during the course of BPD (116).

Endothelin- 1 (ET-1) is a pro-inflammatory and potent vasoconstrictor expressed in high concentrations in the lung. A study evaluated ET-1, IL-6, and IL-8 expression in tracheal aspirates (TAs) from preterm infants with and without BPD, at various time points. The levels of ET-1, IL-6, and IL-8 gradually increased from days 1 to 7 of life in TAs from the BPD group, but not in controls. Additionally, leukocyte counts in TAs were also higher in the BPD group (117).

Together, these studies have identified biomarkers in lung fluids that are likely to be associated with local changes in gene expression. However, the procurement of these samples is more invasive, and requires the patient to be intubated, which limits its use and the availability of proper controls in studies. We believe that these molecules represent not only potential powerful disease diagnostic tools but also therapeutic targets for drugs that act locally in the lung.

#### Genetic Biomarkers

For many decades, investigators have hypothesized that BPD was a genetic disease. Multiple research groups performed cohort, twin, and familial DNA genotyping studies (59, 118–120). However, most studies have failed to identify genetic associations with BPD development and/or severity. One research group examined DNA samples from BPD patients from different populations (Northern Finnish, Canadian, and Hungarian) to compare 44 SNPs in the IL-6, IL-6R, and gp130 (IL-6ST), IL-10, tumor necrosis factor (TNF), and glucocorticoid receptor (NR3C1) genes, but failed to identify associations with BPD susceptibility (121).

A recent genome-wide association study (GWAS) conducted in a large cohort of preterm infants (*n* = 1,091) evaluated the association between SNP and BPD. The investigators genotyped 6,324 SNPs in 601 selected genes in patients with or without RDS and BPD (using the “old” BPD definition). Furthermore, the preterm infants were classified according to their ethnicity in Caucasian (CA; *n* = 922) and African American (AA; *n* = 169) groups, and confirmed their findings in two independent populations. This study identified two SNPs (rs3771150 in the IL-18 gene and rs3771171 in the IL-18RAP gene) associated with BPD development in AA patients. Because these genes are known mediators of interleukin-18 (IL-18) signal transduction pathways, the investigators postulated that IL-18 might play a regulatory role in BPD, probably by altering expression of pro-inflammatory cytokines (78). A second GWAS study in CA and AA patients, conducted by a different group in 418 preterm infants identified polymorphisms in two SNPs of the SPOCK2 gene (rs1245560 and rs1049269) with increased risk for BPD (79). Finally, another study using a combination of GWAS and pathway-based approaches evaluated more than a million SNPs in 751 preterm infants with and without BPD, but failed to identify SNPs associated with the disease that had genome-wide significance. However, pathway analysis performed on the top close-to-significant genes and lower false discovery rates revealed that the miRNA-219 family (involved in resolution of acute inflammation), the phosphorus oxygen lyase activity pathway, and the CD44 gene were associated with BPD and/or death. The study concluded that genetic predisposition to develop severe BPD might involve mutations affecting pathways of lung development and repair (82). Therefore, the development of future therapies will need to consider targeting these genes and pathways in a “personalized genomics” approach.

#### miRNA Biomarkers

The use of miRNAs, a recently discovered class of non-coding RNAs involved in gene expression regulation, as biomarkers has become more common in the last decade (122). This is due to the remarkably high stability of miRNAs in biofluids, as well as the low complexity of the available methods to detect and quantify miRNAs, such as qPCR and RNAseq. A recent study conducted to evaluate miRNA expression in PB obtained from 50 preterm infants identified four miRNAs (miR-133b; miR-7; miR-152; and miR-30a-3p) associated with BPD (80). Another study found downregulation of miR-206 in mouse lung tissue of an experimental BPD model vs. control. This study also found lower blood miR-206 levels in a cohort of BPD patients vs. controls. Moreover, overexpression of miR-206 in lung cells resulted in increased apoptosis, and decreased proliferation, migration, and adhesion, as well as downregulation of the fibronectin gene (FN1) (downregulation of this miRNA resulted in the opposite effect) (81).

In summary, the diagnostic and prognostic potential of miRNAs is related to their high stability, and easy detection by molecular biology approaches. A growing number of studies support a potential use of secreted miRNAs as biomarkers for a wide variety of diseases as well as predictors of drug sensitivity and outcome (53, 123). In diseases, such as lung cancer, detecting elevated expression of single miRNAs in sputum shows a higher sensitivity in diagnosis than sputum cytology analysis (107). Moreover, small sets of miRNAs have already been shown to enable classification of different disease states and conditions (38, 44, 107). For example, miRNA panels can be used to distinguish among different types of lung carcinoma, indicating that miRNAs could potentially serve as robust diagnostic biomarkers of disease subtypes (16, 60, 116, 121). Finally, the use of miRNA as therapeutic tools has reached the clinical phase for various diseases, and continues to expand (17, 79, 118). In the particular case of lung disease, tissue-specific therapeutic modulation of miRNA activity has been achieved via inhalation in animal models (7, 55, 75, 78). Therefore, the use of miRNAs as biomarkers for pediatric inflammatory lung disease may also facilitate the future development of individualized diagnosis and medical strategies for treatment.

### Biomarkers for PH

As seen below, multiple biomarkers have been investigated in the search of an early diagnostic tool for BPD in preterm infants. However, only a few studies have focused in the identification of biomarkers for PH development in patients with BPD. Of these, one study evaluated plasma levels of asymmetric dimethylarginine (ADMA), an inhibitor of the production of NO, as a predictor of PH in preterm infants with BPD (88, 124). The study was conducted in 118 preterm infants with BPD, of which 23 developed PH. In addition to ADMA, arginine, citrulline, ornithine, and proline levels were also analyzed. ADMA concentrations were higher in patients with both BPD and PH than in patients with BPD alone; and plasma levels of arginine, citrulline, ornithine, and proline did not show differences between groups. However, the plasma arginine to ADMA ratio was significantly low in patients that developed PH. The study concluded that ADMA is not only a biomarker of PH in BPD patients but it also represents a potential therapeutic target for the disease (88). Another study evaluated serum levels of endostatin/Angiopoietin-1 (AP-1) in premature infants with severe BPD with and without PH. Both endostatin levels and the ratio of endostatin/AP-1 were significantly increased in infants with severe BPD and PH on day 7 of life, indicating that a high endostatin/AP-1 ratio may lead to abnormal angiogenesis and could serve as a biomarker for PH (125).

Studies have shown that NO plays an important role in the control of neonatal pulmonary vascular resistance. Therefore, genetic studies of NO pathways have evaluated associations of BPD and PH, by studying SNPs in genes of the l-arginine/NO pathway in 140 premature neonates with BPD with and without PH. The study identified a SNP in the arginase I gene (rs2781666) associated with low arginase I levels in the BPD + PH group, suggesting a protection against PH development (89).

Finally, a study conducted in 277 preterm infants evaluated the relationship between early echocardiographic signs of pulmonary vascular disease with the development of BPD or PH. In this study, echocardiogram evaluations were performed at 7 days of age (early) and at 36 weeks PMA (late) to measure right ventricular systolic pressure, cardiac shunt, and ventricular septal wall flattening. Of the 227 infants who developed BPD, early PH was diagnosed in 115 infants (42%), and late PH was identified in 39 patients (14%). The investigators concluded that echocardiogram at day could be used to identify preterm infants with high risk for BPD and PH (6).

In summary, a critical barrier in PH prevention and effective treatment is the lack of a good diagnostic tool to identify BPD patients at risk for PH. Currently, the diagnosis of PH in BPD patients occurs via highly invasive procedures, such as cardiac catheterization. As seen above, only a handful of studies have explored the use of alternative approaches, and the identification of biomarkers for PH detection and prediction in BPD patients. We believe that the standardization of methods for PH diagnosis in pediatric patients will allow the development of larger studies that will explore genetic, protein, and miRNA biomarkers for BPD-associated PH, in several biofluids. Integration of this information will help increase the accuracy of pediatric PH diagnosis in BPD, and will provide new mechanistic information for the future development of therapeutic interventions.

## BPD/PH Management and Available Experimental Therapies

The first step in the management of PH in neonates with BPD is treatment and prevention of severe BPD. However, there continues to be a need to have multi-center controlled trials for the treatment of BPD-associated PH that can identify effective strategies for management and prevention. In all neonates with PH, congenital heart disease must be ruled out, particularly in the setting of persistent pulmonary hypertension of the newborn (PPHN). Pulmonary vein stenosis is a rare cause of PH in neonates and is a close differential diagnosis of PH in older preterm infants with BPD (126). Although pulmonary vasodilators such as iNO, PDE5 inhibitors (sildenafil, tadalafil), prostacyclins, bosetan, and CCB have been used in the treatment of PH in neonates, prevention of PH remains the best treatment. Unfortunately, the US food and drug administration (FDA) remains skeptical in approving some of these medications, because these classes of medications have narrow therapeutic indices, hence posing significant safety issues. “Off label” use is common practice, as most clinical trials are carried out in adults. Another challenge in the treatment of PH in neonates is the ability to clearly define a clinical endpoint and monitoring of clinical improvement. Monotherapy or combination treatment have been used, but their efficacy and their short- and long-term adverse effects are considered prior to instituting therapies for neonatal PH. iNO is currently approved for treatment of PPHN in neonates, but its use in the prevention of BPD and PH in preterm infants remains to be established (127, 128). Sildenafil remains a popular option in the treatment of BPD-associated PH due to its ease of administration, however, its use is off label in children, and there is paucity of data/evidence for its use in preterm infants with BPD/PH (129). Preventive strategies, such as limiting hypoxia and hyperoxia, reducing prolonged mechanical ventilation, and treatment of hemodynamically significant PDA are all important in the treatment/prevention of BPD. Clinicians must pay attention to the growth and nutrition of infants with BPD as lung growth and alveolarization continue in the first 2 years of life, and supporting somatic growth is very critical. Early treatment of intercurrent lung infections is also very important, particularly in infants who are intubated and receiving invasive mechanical ventilation. Furthermore, chronic mechanical ventilation should be considered in infants with severe BPD who cannot be weaned from non-invasive positive pressure support. In such infants, chronic hypoxemic episodes and impaired ventilation together contribute to impaired lung growth and increasing their risks to developing PH. Finally, a fine balance should also be maintained between hypoxemia and hyperoxia; hence, oxygen-saturation target is monitored carefully in older preterm infants with BPD. Hyperoxia potentially can lead to increased oxygen free radicals that can contribute to lung injury.

Animal experimental models have explored the use of mesenchymal stem cells (MSCs) in the treatment of BPD. Studies show that MSCs therapy has paracrine effects with anti-inflammatory and antifibrotic properties that help reduce BPD, by inducing vascular and alveolar growth, and inhibiting lung fibrosis (130). Furthermore, in studies performed on neonatal rats, BPD induced by hyperoxia decreased circulating and resident MSCs in the lung (131). Overall, the available literature indicates that more research is needed on stem cell therapeutic doses, long-term effects, and safety before their use becomes available for the management of BPD.

## Disease Prevention

Despite preterm and VLBW infants having a high risk of BPD, not all of them develop BPD. This implies that BPD could be prevented. According to the NHLBI workshop on the primary prevention of CLDs, the clue for BPD prevention is the identification of molecular pathways that affect normal lung development and lead to BPD (Figure 1). Early identification of the prenatal and antenatal factors that affect these molecular pathways will lead to timely interventions that may prevent endothelial injury and oxidative stress (3). Because maternal smoking has been associated with preterm labor and subsequently BPD development (132), smoking cessation through counseling is one of strategies that can be performed in the preconception stage and early pregnancy. Inhaled corticoids for prevention of BPD have also been evaluated; however, their use is still controversial due to their multiple side effects (133–135). A recent study has reported the use of intratracheal budesonide with surfactant in preterm infants diagnosed with severe RDS to decrease their risk of developing BPD. The results showed a decrease in oxygen requirement, lung injury, and pro-inflammatory cytokine secretion (136). The investigators used surfactant as a carrier in order to reach lung periphery and increase the solubility of budesonide; making it more effective. Furthermore, budesonide remained in the lung for up to 8 h when administered locally, as opposed to shorter times when administered systemically (137).

Several pharmacological interventions have been evaluated in animal models of BPD and PH in search of new molecules and pathways to explain the abnormal lung development in premature newborns with these diseases. As a result, therapeutic molecules, such as caffeine and other xanthine derivatives, vitamin A, and vitamin A analogs, were found to be effective at promoting lung maturation (63). Caffeine treatment has also been used in preterm infants to prevent apnea of prematurity, facilitate extubation, and decrease time on mechanical ventilation, which also lead to decreased BPD (107, 138, 139). However, although caffeine has anti-inflammatories properties, levels of caffeine outside of therapeutic range are associated with an increase in inflammation (140). In addition, a decrease in weight gain has been reported during the three first 3 weeks of caffeine therapy in preterm neonates (141). Vitamin A is another therapeutic agent with high evidence for prevention of BPD in extremely preterm infants (138) that has similar benefits to caffeine in decreasing duration of intubation and oxygen therapy (142).

Studies in animals have found a decrease in soluble guanylate cyclase (sGC) in mouse pups following hyperoxia-induced lung injury, indicating that activation of sGC could be used as a potential strategy for prevention of BPD (143). Another study in rats showed that sildenafil inhibition of phosphodiesterase 5 (PDE5) resulted in increased vascular distribution in lung tissue. Moreover, both hypoxia-inducible factor (HIF) and VEGF levels were higher in lungs of BPD rats, suggesting that HIF could be used for management of BPD (144). Other experimental models of BPD have shown that vitamin D plays important roles in lung maturation, suggesting that vitamin D supplementation could be a modifiable risk factor of BPD (123). Furthermore, abnormal changes in alveolar development, tracheal mesenchymal differentiation markers, and surfactant synthesis were found in the offspring of rats with vitamin D deficiency (145). Finally, studies in mice have suggested that the mother’s diet during pregnancy can cause placental inflammation and affect fetal lung development. In this regard, high-fat diet during pregnancy resulted in alveolar simplification and a reduction of biochemical and structural indicators of lung maturation in mouse pups (146). Therefore, studies linking the composition of the mother’s diet during pregnancy and the development of BPD and other lung diseases of the newborn will warrant the use of dietary recommendations as potential prevention therapies.

## Summary and Conclusion

Bronchopulmonary dysplasia is a multifactorial disease that remains the most serious lung condition of prematurity, and a significant cause of mortality in preterm infants. Early detection and diagnosis of BPD is critical to prevent disease progression and chronic lung remodeling. Infants with BPD are at increased risk for long-term hospitalization, requirement of oxygen supplementation after discharge, recurrent respiratory complications, and lifelong consequences that result from their impaired lung development and disturbed lung growth. Subclinical lung injury driven by incompletely resolved or low-grade chronic inflammation can promote disease processes that manifest as fibrosis, emphysema, COPD, cancer, and interstitial lung disease later in life (147, 148). In addition, between 14 and 38% of BPD patients develop PH, a condition with over 40% mortality that often complicates BPD (5, 6, 149, 150). The association of BPD with PH predisposes neonates to lung and cardiovascular injury, increasing susceptibility to infection and mortality, and affecting lung health throughout adulthood.

While there is currently no effective treatment for BPD, studies have shown that variation in ventilator settings, extubation followed by non-invasive ventilation (e.g., nasal continuous positive airway pressure), together with vitamin A, caffeine, NO, and/or surfactant therapy may improve the outcome of BPD (151–156). Preventive strategies, such as minimization of feeding-related aspiration, optimization of nutrition, strict avoidance to tobacco smoke, and prevention of infection by close adherence to immunization schedules have been proven to minimize lung injury, and help in resolution of BPD (157). Therefore, identification of novel less invasive early biomarkers for neonatal BPD is necessary to improve detection and prognostic outcome of respiratory pathologies, and to initiate proper treatment that will prevent later complications. Moreover, development of individualized medicine to treat inflammatory lung disease has been hindered by our lack of understanding of the mechanisms responsible for inter-individual differences in susceptibility and severity. Thus, characterization of molecular events that result in altered gene expression during the course of lung disease is important to facilitate the development of individualized diagnosis and medical treatments before potentially chronic and dire downstream consequences become irreversible.

Although multiple studies have focused on the identification of genetic and epigenetic markers to predict and prevent BPD, no good predictive genetic markers have been reported to date. More recently, the search for biomarkers has been extended to biofluids, including blood, urine, BALF, TAs, etc., and these studies were successful in identifying associations of biomarkers with disease occurrence and severity. Although the majority of the reported markers are of protein nature, other molecules such as gases, metabolites, and miRNAs were also found associated with BPD and PH. Functional and computational analyses have revealed that these are involved in pathways associated with immunity, inflammation, and oxidative stress. These biomarkers are likely to complement the genetic information in the identification of patients vulnerable to develop BPD and associated diseases, such as PH, and will help in the discovery of molecular mechanisms implicated in the disease pathophysiology.

Many of the biomarkers reported here were linked to disease development and/or severity, and their levels appear to depend on the time point in which the sample was collected, and/or the infant’s age. Moreover, except for some genetic studies conducted in large cohorts, many of the studies reported here were exploratory and performed in a very small number of patients, without validating results in an independent population. We believe that the studies presented here have provided important data on biomarker expression, purification, stability, associations with disease, and other information necessary for the design of reliable predictive tools for BPD diagnosis, severity, and likelihood of response to therapeutics. The work performed over the past 15 years has provided the stepping-stone for the design of novel studies involving more complex techniques, such as next generation sequencing, and more specific and sensitive antibodies for the identification of biomarkers for BPD and PH. The generation of tools for early detection of patients at risk for BPD and PH will warrant the use of more aggressive and personalized treatment with medications, respiratory support, nutrition, and preventive care procedures that will improve disease outcomes.

## Author Contributions

LR designed and organized the manuscript structure, wrote the manuscript, searched and curated the literature, designed figures, and created tables. RS and CO-M searched and curated the literature and wrote the manuscript section on diagnosis and treatment. GS searched and curated the literature, wrote the manuscript, and created figures and tables. PS designed and organized the manuscript structure, searched and curated the literature, designed figures, and coordinated every author’s contributions and roles.

## Conflict of Interest Statement

The authors declare that the research was conducted in the absence of any commercial or financial relationships that could be construed as a potential conflict of interest.
